# Activation of *Salmonella* Typhi-Specific Regulatory T Cells in Typhoid Disease in a Wild-Type *S*. Typhi Challenge Model

**DOI:** 10.1371/journal.ppat.1004914

**Published:** 2015-05-22

**Authors:** Monica A. McArthur, Stephanie Fresnay, Laurence S. Magder, Thomas C. Darton, Claire Jones, Claire S. Waddington, Christoph J. Blohmke, Gordon Dougan, Brian Angus, Myron M. Levine, Andrew J. Pollard, Marcelo B. Sztein

**Affiliations:** 1 Center for Vaccine Development, University of Maryland School of Medicine, Baltimore, Maryland, United States of America; 2 Department of Epidemiology and Public Health, University of Maryland School of Medicine, Baltimore, Maryland, United States of America; 3 Oxford Vaccine Group, Department of Paediatrics, University of Oxford and the National Institute for Health Research Oxford Biomedical Research Centre, Oxford, United Kingdom; 4 Microbial Pathogenesis Group, Wellcome Trust Sanger Institute, Hinxton, United Kingdom; 5 Nuffield Department of Medicine, University of Oxford, United Kingdom; Stanford University School of Medicine, UNITED STATES

## Abstract

*Salmonella* Typhi (*S*. Typhi), the causative agent of typhoid fever, causes significant morbidity and mortality worldwide. Currently available vaccines are moderately efficacious, and identification of immunological responses associated with protection or disease will facilitate the development of improved vaccines. We investigated *S*. Typhi-specific modulation of activation and homing potential of circulating regulatory T cells (T_reg_) by flow and mass cytometry using specimens obtained from a human challenge study. Peripheral blood mononuclear cells were obtained from volunteers pre- and at multiple time-points post-challenge with wild-type *S*. Typhi. We identified differing patterns of *S*. Typhi-specific modulation of the homing potential of circulating T_reg_ between volunteers diagnosed with typhoid (TD) and those who were not (No TD). TD volunteers demonstrated up-regulation of the gut homing molecule integrin α4ß7 pre-challenge, followed by a significant down-regulation post-challenge consistent with T_reg_ homing to the gut. Additionally, *S*. Typhi-specific T_reg_ from TD volunteers exhibited up-regulation of activation molecules post-challenge (e.g., HLA-DR, LFA-1). We further demonstrate that depletion of T_reg_ results in increased *S*. Typhi-specific cytokine production by CD8+ T_EM_ *in vitro*. These results suggest that the tissue distribution of activated T_reg_, their characteristics and activation status may play a pivotal role in typhoid fever, possibly through suppression of *S*. Typhi-specific effector T cell responses. These studies provide important novel insights into the regulation of immune responses that are likely to be critical in protection against typhoid and other enteric infectious diseases.

## Author Summary

In this manuscript, we describe, for the first time, a potential role for regulatory T cells (T_reg_) as an important factor in determining disease outcome in humans following exposure to wild-type *S*. Typhi. We studied in considerable depth the modulation of T_reg_ activation characteristics and their homing potential in the development of typhoid disease following a wild-type *S*. Typhi challenge in a unique human infection model. We show that *S*. Typhi-specific up-regulation of the gut homing molecule integrin α4β7 pre-challenge is associated with subsequent development of typhoid disease. We further demonstrate that increased *S*. Typhi-specific expression of molecules associated with T_reg_ activation as well as distinct kinetics of the expression of key activation molecules involved in T_reg_ function are present in volunteers diagnosed with typhoid disease. We also provide the first evidence that T_reg_ can functionally suppress *S*. Typhi-specific CD8+ T cells *in vitro*. These intriguing results suggest that T_reg_ are likely to play a role in the development of typhoid fever and potentially other enteric infections.

## Introduction

*Salmonella enterica* serovar Typhi (*S*. Typhi), the causative agent of typhoid fever, is a major public health threat throughout the developing world. An estimated 26.9 million cases resulting in approximately 217,000 deaths occur annually [1,2]. Furthermore, antibiotic resistance increasingly limits treatment options in many areas [3,4]. Current typhoid vaccines licensed in the US provide modest protection and are only moderately immunogenic [5,6]. In order to effectively develop new vaccine candidates that will provide robust, long-lasting protection, an improved understanding of the immune correlates of protection is desirable. The recent re-establishment of the human challenge model with wild-type *S*. Typhi provides a unique opportunity to investigate in detail the immune responses following exposure to this pathogen [7].

While multiple studies have investigated cell-mediated immune (CMI) responses against *S*. Typhi immunization and infection [8,9,10,11,12,13,14,15], to date there are no published studies of the potential role of regulatory T cell (T_reg_) responses against this organism. T_reg_ are a specialized subset of CD4+ T cells that are responsible for regulating other immune cells [16,17,18]. They are characterized by expression of interleukin (IL)-2 receptor α (CD25) and the transcription factor Forkhead box protein (Fox)P3 [18]. T_reg_ may be derived *in vivo* in the thymus (tT_reg_) or the periphery (pT_reg_) as well as following *in vitro* activation (iT_reg_) [19]. At present, there is considerable controversy regarding the expression of specific molecules (e.g., Helios for tT_reg_) that enable the distinction among these subsets [19]. For simplicity, since we did not measure expression of the considerable number of molecules required to potentially differentiate among these subsets, in the current studies we refer to circulating T_reg_ as those which were obtained *ex vivo* from the peripheral blood and are likely to represent a combination of tT_reg_ and pT_reg_. Activated T_reg_ may traffic to the sites of specific immune responses and exert their regulatory functions via cytotoxic T-lymphocyte-associated protein 4 (CTLA-4; CD152) competition for co-stimulatory molecules (CD80 and CD86) on antigen presenting cells, consumption of IL-2, and production of suppressive cytokines [17]. Alterations in homing molecules/chemokine receptors expressed by T_reg_ affect their ability to traffic to the site of specific immune responses [16,20,21,22,23]. In addition to their roles in autoimmunity and cancer biology, T_reg_ have been shown to play a role in suppression of immune responses against multiple pathogens, potentially contributing to disease [24,25].

In the present studies we have evaluated the characteristics and kinetics of T_reg_ homing potential and activation, as well as the functional capacity of T_reg_ to suppress *S*. Typhi-specific T cell responses following wild-type challenge of healthy adult volunteers. Of importance, we identified distinct homing potential and activation patterns associated with typhoid diagnosis indicating that T_reg_ may play an important role in the development of typhoid fever. In fact, it is likely that immune homeostasis between suppressive and inflammatory responses is critical to the prevention of disease. These studies describe for the first time, the role of *S*. Typhi-specific modulation of T_reg_ homing potential and activation characteristics in typhoid disease, a role which may be broadly applicable to other enteric infections.

## Results

### Similar levels of circulating T_reg_ and *ex vivo* T_reg_ proliferation were observed in volunteers diagnosed with typhoid (TD) and those who were not (No TD)

**Fig 1:**
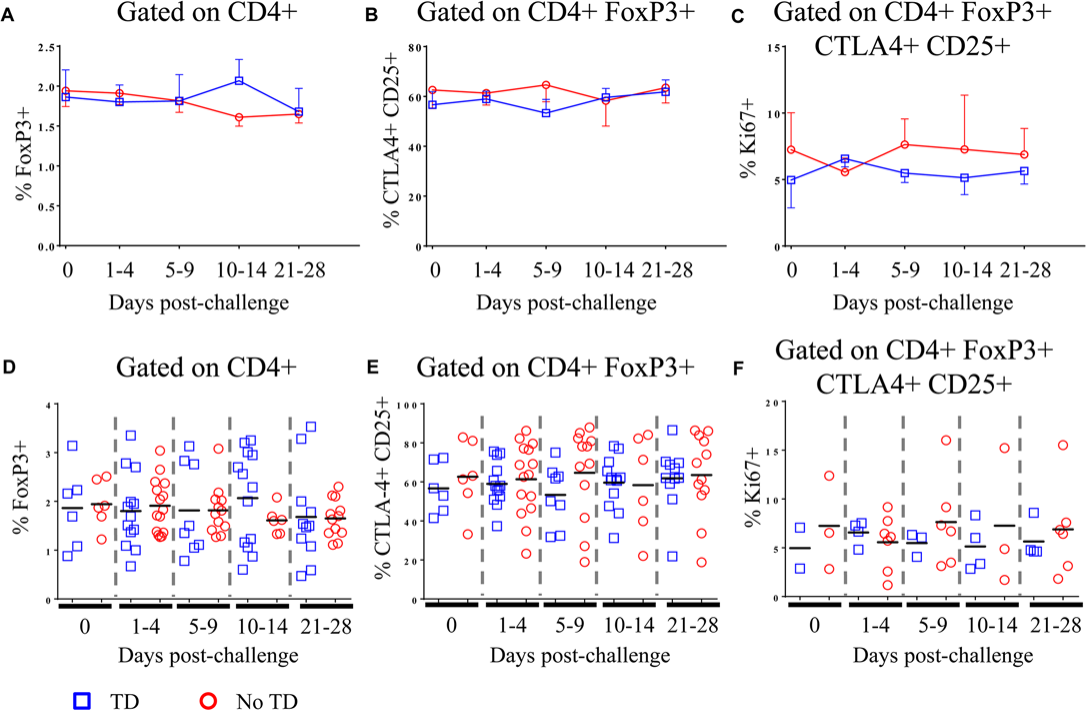
Percentages of circulating and proliferating T_reg_ in TD and No TD volunteers. **A)** Percentage of CD4+ T cells positive for FoxP3 expression and **B)** percentage of CD4+ FoxP3+ T cells positive for CTLA-4 and CD25 in PBMC obtained pre-challenge and at multiple time-points after challenge (TD n = 6; No TD n = 6). **C**) Percentage of CD4+ FoxP3+ CTLA-4+ CD25+ T cells expressing Ki67 (a marker of proliferation) *ex vivo* (TD n = 2, No TD n = 3). Values are shown as the mean +/- SEM. Scatter plots showing **D)** the percentage of CD4+ T cells positive for FoxP3 expression and **E)** the percentage of CD4+ FoxP3+ T cells positive for CTLA-4 and CD25 in PBMC obtained pre-challenge and at multiple time-points after challenge (TD n = 6; No TD n = 6). **F**) the percentage of CD4+ FoxP3+ CTLA-4+ CD25+ T cells expressing Ki67 (a marker of proliferation) *ex vivo* (TD n = 2, No TD n = 3). Means are indicated with a black horizontal line. P-values were determined using a mixed effects regression model. TD (blue squares); No TD (red circles). Values from multiple time-points were grouped together in time segments (1–4, 5–9, 10–14, and 21–28 days post-challenge) to account for variability in the samples available from each volunteer. Some volunteers had samples from multiple time-points in a time-segment resulting in more data points than the corresponding number of volunteers.

Peripheral blood mononuclear cells (PBMC) from healthy adult volunteers were obtained prior to and at multiple time-points following challenge with ~2 x 10^4^ colony forming units (cfu) of wild-type *S*. Typhi (**S1 Fig**) [7]. Volunteers who developed a fever ≥38°C sustained for ≥12 hours and/or blood culture-confirmed *S*. Typhi bacteremia were diagnosed with typhoid as described [7]. In the present study, randomly selected volunteers meeting criteria for typhoid diagnosis (TD, n = 6) and volunteers who did not meet criteria (No TD, n = 6) were assessed for T_reg_ phenotype, activation status, and homing potential. In the randomly selected TD volunteers, the time of typhoid diagnosis ranged from 6–9 days post-challenge. Flow cytometry was used to detect the percentages of circulating CD4+ FoxP3+ T_reg_ as well as the more stringently defined CD4+ FoxP3+ CTLA-4+ CD25+ T_reg_ subset in unstimulated PBMC. The gating strategy is shown in **S2 Fig**. Percentages of FoxP3+ cells ranged from <1% to 3.5% of total CD4+ T cells, a finding consistent with previous reports [26,27]. CTLA-4+ CD25+ T_reg_ ranged from 23–88% of CD4+ FoxP3+ T_reg_. There was considerable variation among volunteers in both groups and we found no significant difference between TD and No TD volunteers in the pre- or post-challenge percentages of circulating T_reg_ as defined by either strategy (**Fig 1A, 1B, 1D and 1E**). Furthermore, no statistically significant differences in the percentage of circulating T_reg_ over time were noted in either group (**Fig 1A, 1B, 1D and 1E**). Furthermore, in a subset of volunteers, we measured Ki67 expression *ex vivo* as a surrogate of proliferation. While circulating T_reg_ expressed Ki67 indicating that a small proportion of them were proliferating *in vivo*, there was no difference in the magnitude or kinetics of Ki67 expression between TD and No TD volunteers (**Fig 1C and 1F**).

### Differential expression of homing molecules in circulating T_reg_ from TD and No TD volunteers

Although no differences were identified between TD and No TD volunteers in the total percentage of circulating T_reg_, we hypothesized that differences in the *S*. Typhi-specific modulation of the homing potential of T_reg_ might be associated with typhoid diagnosis. To evaluate this possibility, PBMC from volunteers challenged with wild-type *S*. Typhi (as described above) were stimulated with *S*. Typhi-infected autologous Epstein Barr Virus (EBV)-transformed B lymphoblastoid cell lines (B-LCL) or non-infected B-LCL (negative control). Flow cytometry was utilized to detect expression of the gut homing molecule integrin α4β7, as well as CXCR3 (homing to sites of inflammation) and CCR6 (homing to sites of T_H_17 inflammation). Relative (net) *S*. Typhi-specific modulation of the expression of homing molecules was determined by subtracting the values obtained following stimulation with non-infected B-LCL from stimulation with *S*. Typhi-infected B-LCL.

**Fig 2:**
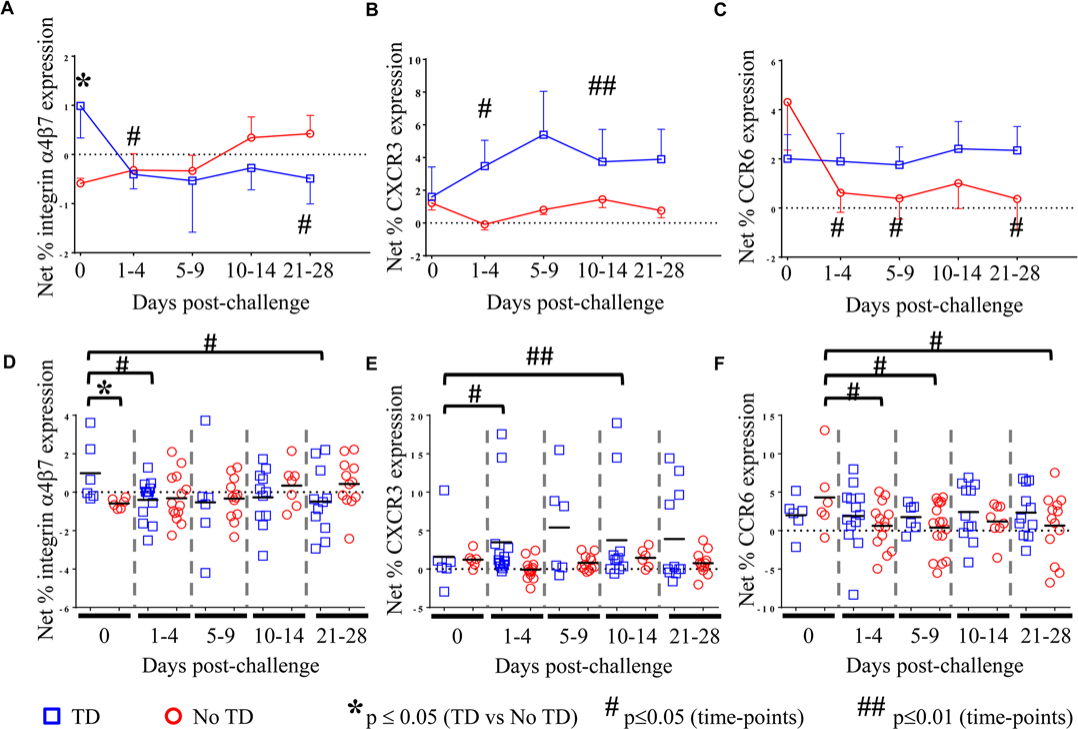
*S*. Typhi-specific homing potential of circulating T_reg_. Net *S*. Typhi-specific expression of **A**) integrin 47, (TD n = 6, No TD n = 6) **B)** CXCR3, (TD n = 6, No TD n = 6) **C)** CCR6 (TD n = 6, No TD n = 6) Values are shown as the mean +/- SEM. Scatter plots showing the net expression of **D)** integrin α4β7, **E)** CXCR3, and **F)** CCR6 on *S*. Typhi-specific T_**reg**_. Means are indicated with a black horizontal line. Time points with statistically significant differences between TD and No TD volunteers (*) or among time-points within each group (#) are identified. P-values were determined using a mixed effects regression model. TD (blue squares); No TD (red circles). Values from multiple time-points were grouped together in time segments (1–4, 5–9, 10–14, and 21–28 days post-challenge) to account for variability in the numbers of samples available from each volunteer. Some volunteers had samples from multiple time-points in a time-segment resulting in more data points than the corresponding number of volunteers.

As an enteric pathogen, the gut is the first site of immune encounter with *S*. Typhi, and therefore, we conjectured that the ability of *S*. Typhi to up-regulate the expression of integrin α4β7 (a gut homing molecule) on circulating T_reg_ might be associated with suppression of protective host responses contributing to disease. We observed that pre-challenge levels of *S*. Typhi-specific modulation of integrin α4β7 expression were indeed higher on circulating T_reg_ isolated from volunteers who were subsequently diagnosed with typhoid (TD; p = 0.054—mixed effects regression model (**Fig 2A and 2D**)). Of note, *S*. Typhi-specific expression of integrin α4β7 was significantly down-regulated in TD volunteers in the 1–4 day time-frame after challenge (p = 0.047—mixed effects regression model), remaining at relatively stable levels thereafter (**Fig 2A and 2D**). Interestingly, there was an opposite trend in No TD volunteers with up-regulation of *S*. Typhi-specific integrin α4β7 expression in the 10–14 and 21–28 days post-challenge time frames; however, these changes did not reach statistical significance.

We further investigated whether differences in CXCR3 expression are able to distinguish TD and No TD volunteers as *S*. Typhi-specific T_reg_ homing to sites of active inflammation could also suppress a protective immune response. Both TD and No TD volunteers exhibited *S*. Typhi-specific modulation of CXCR3 expression prior to challenge; however, there was no difference observed between the groups (**Fig 2B and 2E**). Following challenge, CXCR3 expression was significantly up-regulated on *S*. Typhi-specific T_reg_ in TD volunteers (p = 0.04—mixed effects regression model) with 2 volunteers showing particularly high levels of *S*. Typhi-specific up-regulation of CXCR3 expression (**Fig 2E**). Expression of CXCR3 upon exposure to *S*. Typhi-infected targets remained relatively constant over time in No TD volunteers.

Additionally, because T_C_17 responses have been identified following Ty21a immunization [8], we hypothesized that expression of CCR6 may also lead to homing of T_reg_ to sites of *S*. Typhi-induced inflammation. While *S*. Typhi-specific modulation of the expression of CCR6 by circulating T_reg_ was detected, we did not find significant differences between TD and No TD volunteers prior to challenge (**Fig 2C and 2F**). *S*. Typhi-specific up-regulation of CCR6 expression by circulating T_reg_ remained relatively constant in TD volunteers with no significant differences in the mean pre-challenge expression compared to later time-points. In contrast, a significant decrease in *S*. Typhi-specific expression of CCR6 by circulating T_reg_ was evidenced after challenge in No TD volunteers (p = 0.02–0.03—mixed effects regression model) (**Fig 2C and 2F**). Of interest, no differences were detected in *S*. Typhi-specific modulation of CCR6 expression between TD and No TD volunteers following challenge.

### Activation of peripheral *S*. Typhi-specific T_reg_ after challenge is associated with typhoid diagnosis

We hypothesized that increases in *S*. Typhi-specific expression of additional activation molecules on circulating T_reg_ might be associated with suppression of protective responses resulting in typhoid diagnosis. To test this hypothesis, modulation of *S*. Typhi-specific expression levels of activation molecules, including HLA-DR, CD11a (lymphocyte function-associated antigen-1; LFA-1), T cell immunoglobulin mucin (Tim)-3, CD304 (neuropilin-1; NRP-1), CD279 (programmed cell death-1; PD-1), CD27, and CD39 (ectonucleoside triphosphate diphosphohydrolase 1; ENTPD1) on T_reg_ were measured using flow cytometry. As for the measurement of homing potential, PBMC from volunteers challenged with wild-type *S*. Typhi were stimulated with *S*. Typhi-infected autologous B-LCL or non-infected B-LCL. Relative (net) *S*. Typhi-specific modulation of the expression of the activation molecules listed above was determined by subtracting the values obtained following stimulation with non-infected B-LCL from stimulation with *S*. Typhi-infected B-LCL.

**Fig 3:**
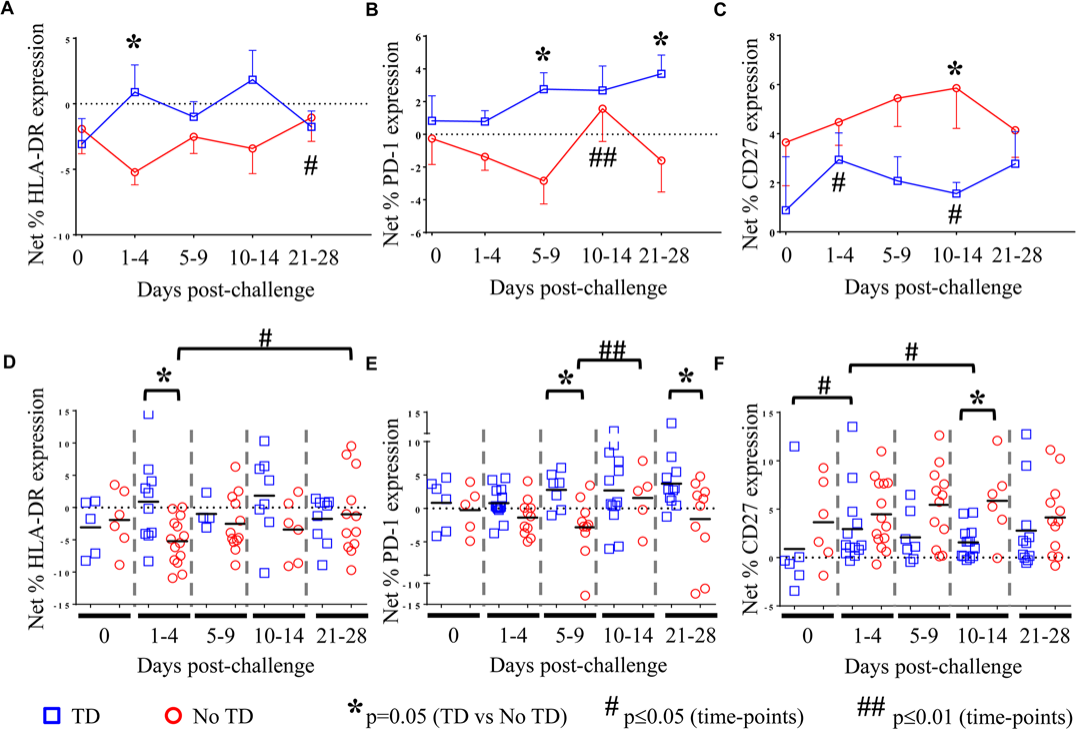
*S*. Typhi-specific activation of circulating T_reg_. Net *S*. Typhi-specific expression of **A**) HLA-DR, (TD n = 5, No TD n = 4) **B**) PD-1, (TD n = 6, No TD n = 6) and **C**) CD27, (TD n = 6, No TD n = 6) on T_**reg**_. Values are shown as the mean +/- SEM. Scatter plots showing the net expression of **D)** HLA-DR, (TD n = 5, No TD n = 4) **E**) PD-1, (TD n = 6, No TD n = 6) and **F**) CD27, (TD n = 6, No TD n = 6) on *S*. Typhi-specific T_**reg**_. Means are indicated with a black horizontal line. Time points with statistically significant differences between TD and No TD volunteers (*) or among time-points within each group (#) are identified. P-values were determined using a mixed effects regression model. TD (blue squares); No TD (red circles). Values from multiple time-points were grouped together in time segments (1–4, 5–9, 10–14, and 21–28 days post-challenge) to account for variability in the numbers of samples available from each volunteer. Some volunteers had samples from multiple time-points in a time-segment resulting in more data points than the corresponding number of volunteers.

**Fig 4:**
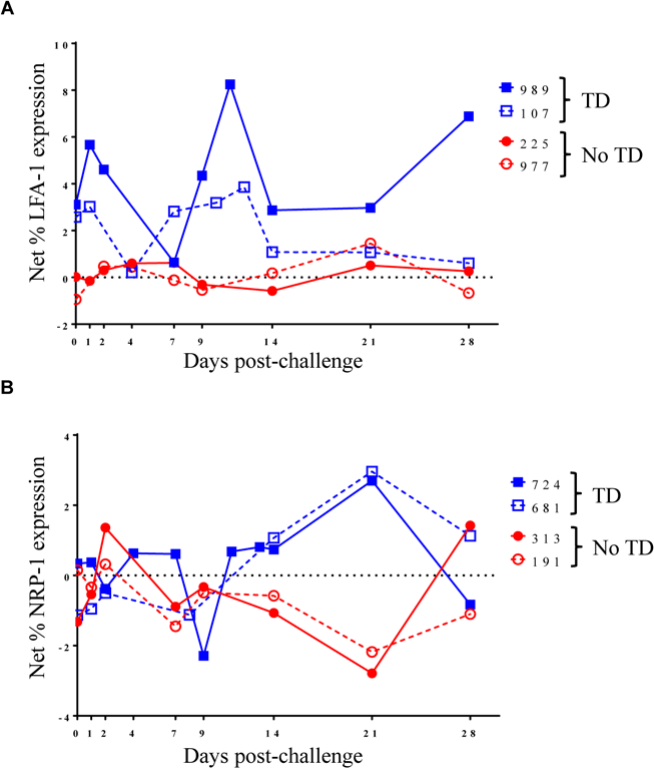
Kinetics of S. Typhi-specific modulation of LFA-1 and NRP-1 expression on circulating T_reg_ following challenge. Kinetic curves from representative volunteers showing net *S*. Typhi-specific modulation of the expression of **A)** LFA-1 and **B)** NRP-1 from day 0 (pre-challenge) until day 28 post-challenge.

There was considerable variability among volunteers in the *S*. Typhi-specific modulation of the expression of all activation molecules. We observed that *S*. Typhi-specific up-regulation of PD-1, CD27, LFA-1, NRP-1, and Tim-3 was present before challenge in many volunteers (**Fig 3B, 3C, 3E and 3F, Fig 4A and 4B and S3B Fig**). However, we identified no significant differences in the *S*. Typhi-specific expression of activation molecules prior to challenge in TD volunteers compared with No TD volunteers (**Fig 3A and 3F, and S3 Fig**). At early time-points (days 1–4) following challenge, however, we observed a notable increase in *S*. Typhi-specific expression of HLA-DR resulting in significantly higher expression in TD than in No TD volunteers (p = 0.015—mixed effects regression model) (**Fig 3A and 3D**). In contrast, *S*. Typhi-specific HLA-DR expression on circulating T_reg_ in No TD volunteers decreased slightly after challenge (days 1–4), returning to baseline levels by 21–28 days post-challenge (**Fig 3A and 3D**).

We also identified marked up-regulation of the expression of PD-1 by *S*. Typhi-infected targets in circulating T_reg_ isolated from TD compared to No TD volunteers post-challenge (**Fig 3B and 3E**). *S*. Typhi-specific up-regulation of PD-1 expression increased gradually in TD volunteers following challenge with the highest levels identified on days 21–28 post-challenge (**Fig 3B and 3E**). Distinctly, however, among those volunteers who did not develop disease we noted a general down-regulation of *S*. Typhi-specific PD-1 expression in circulating T_reg_ 1–9 days post-challenge with a significant increase between the days 5–9 and days 10–14 post-challenge time groups (**Fig 3B and 3E**). *S*. Typhi-specific PD-1 expression returned to baseline levels by days 21–28 post-challenge (**Fig 3B and 3E**). These opposite trends resulted in significantly higher up-regulation of *S*. Typhi-specific expression of PD-1 in circulating T_reg_ in TD compared to No TD volunteers at days 5–9 (p = 0.0097—mixed effects regression model) and 21–28 post-challenge (p = 0.0092—mixed effects regression model) (**Fig 3B and 3E**).

Interestingly, we observed increased up-regulation of the expression of CD27 on *S*. Typhi-specific T_reg_ in No TD compared to TD volunteers (**Fig 3C and 3F**). While the trend was present at most time-points, this difference was statistically significant only in the day 10–14 time frame (after typhoid diagnosis and initiation of antibiotics), (p = 0.031—mixed effects regression model). In a subset of volunteers, *S*. Typhi-specific CD39 expression was also measured. Although there were only a small number of samples tested, we identified up-regulation of *S*. Typhi-specific CD39 expression on circulating T_reg_ following challenge in TD volunteers. This increase peaked at days 10–14 post-challenge and was significantly higher than pre-challenge (p = 0.02—mixed effects regression model) (**S3A Fig**). While *S*. Typhi-specific Tim-3 expression was present on circulating T_reg_ there was no difference noted between TD and No TD volunteers or in either group over time (**S3B Fig**).

### Differential kinetics of activation of circulating *S*. Typhi-specific T_reg_ between TD and No TD volunteers

To further explore changes in *S*. Typhi-specific modulation of the expression of activation molecules over time, we examined kinetic curves of individual volunteers. While significant differences were not detected in the mean expression of LFA-1 between TD and No TD volunteers, the kinetic patterns were remarkably different in volunteers diagnosed, or not, with typhoid following challenge. Despite considerable variation among volunteers, a pattern of increased expression of LFA-1 around the time of disease was identified in a majority of TD volunteers (4/5) while expression remained relatively constant for most No TD volunteers (**Fig 4A**). We also identified differences in the kinetic patterns of *S*. Typhi-specific NRP-1 expression. Unlike LFA-1, we observed *S*. Typhi-specific up-regulation of NRP-1 expression in TD volunteers after diagnosis and initiation of antibiotics (days 14–21 post-challenge) (**Fig 4B**).

### Circulating T_reg_ suppress *S*. Typhi-specific T_eff_ responses

**Fig 5:**
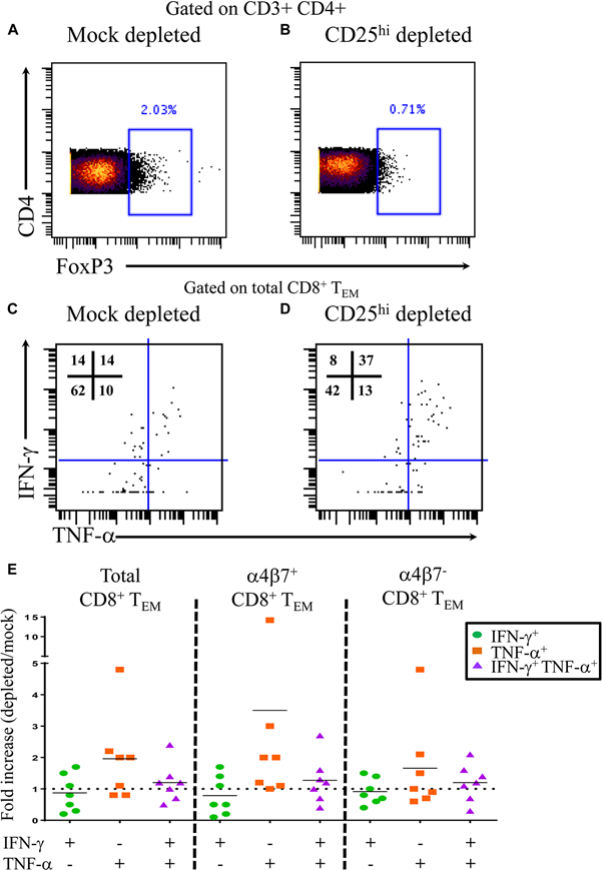
*S*. Typhi-specific cytokine production following T_reg_ depletion. Percentage of CD4+ FoxP3+ T cells following **A)** mock depletion or **B)** CD25 depletion in a representative volunteer. Production of IFN-γ and/or TNF-α by *S*. Typhi-specific CD8+ T_**EM**_ following **C)** mock or **D)** CD25 depletion. **E)** Data are presented as fold increases in IFN-γ and/or TNF-α production by *S*. Typhi-specific total CD8+ T_**EM**_ in depleted vs mock-depleted cultures, as well as by CD8+ T_**EM**_ expressing, or not, integrin α4β7.

To further assess the functionality of T_reg_ in the setting of typhoid disease, we performed CD25 depletion assays. PBMC from 4 TD volunteers were either mock depleted (pan anti-mouse IgG) or CD25 depleted (anti-human CD25) using magnetic bead separation. Time-points were selected based on known *S*. Typhi-specific cytokine responses. A total of 7 independent volunteer-time points were used for depletion studies. Depletion resulted in a 55–74% reduction in FoxP3+ CD4+ T cells (**Fig 5A and 5B**). Following stimulation with *S*. Typhi-infected B-LCL, CD8+ T effector memory (T_EM_) were evaluated for *S*. Typhi-specific cytokine production in the presence (mock-depleted) or absence (CD25-depleted) of T_reg_ using mass cytometry. We found higher percentages of IFN-γ and TNF-α single cytokine producing and multi-functional (IFN-γ+ TNF-α+) *S*. Typhi-specific CD8+ T_EM_ when T_reg_ were depleted (**Fig 5C and 5E**). Interestingly, increases in cytokine production were observed in *S*. Typhi-specific CD8+ T_EM_ with or without gut homing potential (integrin α4β7+ and integrin α4β7-, respectively) (**Fig 5E**).

## Discussion

Effective immune responses must balance the need for pathogen-specific inflammatory responses to fight infection with the need to protect the host from the consequences of excessive inflammation. Homeostasis between regulatory and effector T cells is a major component of this balance. T_reg_ suppress T_eff_ by multiple mechanisms including contact dependent mechanisms, such as CTLA-4, as well as contact-independent mechanisms such as IL-10 production. We aimed to investigate the characteristics, kinetics, and functionality of T_reg_ responses in an *S*. Typhi human controlled infection model. Homing of T_reg_ to sites of specific inflammation has been previously shown [16,20,21,22,23]. Integrin α4β7 is an important molecule associated with homing of lymphocytes to the gut, the site of initial encounter with *S*. Typhi [28]. Here we identified, for the first time, significantly higher pre-challenge gut homing potential of circulating T_reg_ (up-regulation of *S*. Typhi-specific integrin α4β7 expression) in volunteers who were subsequently diagnosed with typhoid disease compared to those who were not. Following challenge, however, there was a significant decrease in *S*. Typhi-specific integrin α4β7 expression on circulating T_reg_, suggesting that these T_reg_ left the peripheral blood, presumably as a result of homing to the gut microenvironment. It is currently unclear why *S*. Typhi-specific T_reg_ expressing differential levels of integrin α4β7 were observed among volunteers before challenge. Participants were recruited in a non-endemic area and are, therefore, unlikely to have previously encountered *S*. Typhi. However, the *S*. Typhi genome has a high degree of homology with other Enterobacteriaceae. Thus, differences in baseline T_reg_ responses could be the result of previous encounters with other enteric Gram negative bacilli, including those present in the normal gut microbiota. We have previously reported that oral immunization of volunteers with attenuated oral *S*. Typhi vaccines elicits *S*. Typhi-specific T_EM_ which expressed, or not, the gut homing molecule integrin α4β7 [11,29]. It has been shown that T cells activated in the gut preferentially express high levels of integrin α4β7 compared to T cells primed in peripheral lymph nodes [30]. Therefore, it is possible that T_reg_ initially primed in the gut would express higher levels of integrin α4β7 upon re-stimulation resulting in recirculation to the site of initial antigen encounter. It is thus reasonable to speculate that higher levels of T_reg_ homing to the gut may suppress local T_eff_ responses resulting in ineffectual control of the infection ultimately leading to typhoid diagnosis. This hypothesis is further supported by our findings showing the capacity of T_reg_ to suppress *S*. Typhi–specific responses by integrin α4β7+ T_EM_ elicited in volunteers following exposure to wild-type *S*. Typhi. Of note, we have also observed in these volunteers, that T_reg_ suppress *S*. Typhi–specific responses by integrin α4β7- T_EM_, suggesting that specific T_reg_ might also exert their regulatory activity at systemic sites.

In addition to early homing to the gut, we identified *S*. Typhi-specific up-regulation of the expression of both CCR6 and CXCR3. CXCR3 expression on T_reg_ is associated with homing to sites of T_H_1/T_C_1 inflammation [21]. It is known that immunization with *S*. Typhi vaccines, as well as natural infection with *S*. Typhi, induce predominantly T_H_1/T_C_1 type responses [8,9,10,11,12,13,14,15,29]. While not significant, there was a trend toward higher levels of *S*. Typhi-specific up-regulation of CXCR3 expression on circulating T_reg_ in TD volunteers compared to No TD volunteers. However, this was primarily driven by two volunteers. Future studies with additional volunteers will help to establish the validity of these findings. CCR6 is responsible for homing of lymphocytes, including T_reg_, to sites of T_H_17/Tc17 inflammation [20,22]. We have previously identified *S*. Typhi-specific production of IL-17A by CD8+ T_EM_ following Ty21a immunization [8]. While no significant differences were noted in *S*. Typhi-specific up-regulation of CCR6 expression on circulating T_reg_ in TD versus No TD volunteers prior to or following challenge, No TD volunteers exhibited a significant *S*. Typhi-specific decrease in the levels of expression of CCR6 on T_reg_ following challenge, then remained at relatively constant levels through day 28 post challenge. These results suggest that suppression of *S*. Typhi-specific T_H_17/Tc17 responses plays a role in protection from typhoid disease. T_H_17/Tc17 responses are known to produce inflammation including recruitment of neutrophils [31] and increased T_H_17 infiltration has been identified in gut inflammatory conditions, such as in Crohn’s disease [32]. It is possible that excessive inflammation could result in increased gut permeability and subsequent dissemination of *S*. Typhi. It is important to note however, that T_H_17 cells also play an important role in gut mucosal integrity [33]. Therefore, it is likely that the balance of T_reg_ and T_H_17/Tc17 effector responses may be critical in determining disease outcome.

Taken together, these results highlight the likely importance of T_reg_ localization in the development of typhoid fever. Interestingly, integrin α4β7 is the only molecule measured which showed significant pre-challenge differences in *S*. Typhi-specific expression on circulating T_reg_ between TD and No TD volunteers, highlighting the potential importance of the local responses early in infection.

In addition to homing to appropriate sites, the activation status of T_reg_ is likely to affect their potential to suppress *S*. Typhi-specific inflammatory responses. Expression of the activation molecule HLA-DR has been associated with increased contact-dependent activity of human T_reg_ [34]. Furthermore, it has been shown that HLA-DR+ T_reg_, while more active, are also more susceptible to apoptosis [35]. We identified significant increases in HLA-DR expression on circulating *S*. Typhi-specific T_reg_ in TD compared to No TD volunteers in the early time-points (day 1–4) post-challenge suggesting that increased *S*. Typhi-specific activation of circulating T_reg_ may play a role in the development of typhoid fever. Furthermore, we also identified significant differences in the intracellular PD-1 content in *S*. Typhi-specific circulating T_reg_ between TD and No TD volunteers. While only a small percentage of natural T_reg_ express PD-1 on the surface, higher levels of PD-1 transcript have been associated with suppressive function, suggesting that PD-1 expression is also involved in the development of typhoid fever [36]. Similarly, we observed up-regulation of *S*. Typhi–specific surface expression of CD39 in TD volunteers, albeit at later time points. T_reg_ expression of CD39 has been associated with suppression of T_H_17/T_C_17 responses [37] which, as previously mentioned, have been identified following *S*. Typhi immunization [8]. It is therefore possible that CD39 expressing T_reg_ in TD volunteers exert their activity, at least in part, by modulating T_H_17/T_C_17 responses. In contrast, we did not identify differences in *S*. Typhi-specific Tim-3 expression between TD and No TD volunteers. The fact that Tim-3 expression on T_reg_ has been associated with increased suppressive T_reg_ activity [38], but no differences were observed between TD and No TD, suggests that the mechanism(s) of T_reg_ activation may vary depending on the model studied. The observations that increased levels of T_reg_ activation appear to play a role in the development of typhoid fever are supported by the results of depletion studies that show increased *S*. Typhi-specific cytokine production by T_EM_ following depletion of T_reg_.

CD27 has been proposed as a marker for T_reg_ suppressive activity as well as a marker for CD4+ T memory phenotype [39,40]. We identified higher levels of *S*. Typhi-specific CD27 expression in No TD volunteers, particularly at days 10–14 post-challenge. This is in striking contrast to other markers of activation which were all increased in TD volunteers. Both CD27+ and CD27- populations displaying suppressive characteristics have been identified in expanded human T_reg_ [41]. Interestingly, CD27+ T_reg_ were predominantly CD62L+ compared to CD27- T_reg_ suggesting that CD27+ T_reg_ may be localizing to peripheral lymph nodes [41]. Therefore, the tissue distribution of activated T_reg_, their characteristics and levels of activation may constitute important determining factors in protection from typhoid fever by contributing to an appropriate balance between suppressive and inflammatory responses.

In addition to *S*. Typhi-specific increase in HLA-DR expression and up-regulation of PD-1 and CD39 in T_reg_, we also identified differences in the kinetic patterns of other molecules associated with T_reg_ activation including LFA-1 and NRP-1. LFA-1 plays an important role in the formation of T_reg_ aggregates that block access of responder T cells to dendritic cells [18]. In contrast, the precise function of NRP-1 up-regulation on human T_reg_ remains to be elucidated and in some studies NRP-1 expression was not identified on human T_reg_ [42]. However, other studies have shown NRP-1 up-regulation to be associated with T_reg_ activation in humans [43]. Furthermore, in mice, NRP-1 has been suggested as a marker for tT_reg_; however, this has not been definitively shown in humans [19,42]. Here we identified *S*. Typhi-specific up-regulation of NRP-1 in TD volunteers and report differences in the observed kinetics of *S*. Typhi-specific NRP-1 expression between TD and No TD volunteers. The difference in kinetics of *S*. Typhi-specific expression of both LFA-1 and NRP-1 molecules in TD compared to No TD volunteers may indicate that multiple mechanisms of increased T_reg_ activation play a role in T_reg_ responses following *S*. Typhi challenge. Furthermore, these findings suggest that not only the precise balance, but also the timing of T_reg_ responses with inflammatory responses might ultimately determine disease outcome.

While there are no animal models for typhoid fever that fully recapitulate human disease, there have been studies in mice using infection with *S*. Typhimurium which reveal a potential role for T_reg_ in suppressing specific T_eff_ responses [44]. In this mouse model, increased T_reg_ suppressive capacity, including upregulated CTLA-4 (CD152) expression, is associated with higher *S*. Typhimurium bacterial burden. Furthermore, T_reg_ ablation results in enhanced T_eff_ activation leading to reduced pathogen burden. These results support our findings that increased T_reg_ activation is associated with typhoid disease and that T_reg_ are capable of suppressing *S*. Typhi-specific T_eff_ in humans.

In summary, we have shown that *S*. Typhi-specific up-regulation of the gut homing molecule integrin α4β7 prior to challenge is associated with typhoid diagnosis. Moreover, despite differences in the kinetics of the responses among various T_reg_ activation molecules, with the notable exception of CD27, there was a clear trend for circulating T_reg_ from TD volunteers to display increased levels of *S*. Typhi-specific activation. Of great importance, we have also demonstrated that T_reg_ are functionally capable of suppressing *S*. Typhi-specific CD8+ T_EM_ cytokine responses. While the small sample size is a limitation, these studies provide an important first description of T_reg_ responses following *S*. Typhi exposure in humans. Further investigation into how these responses may relate to protection following immunization with attenuated strains of *S*. Typhi will provide much needed information to inform and accelerate the development of novel vaccines for typhoid and other enteric fevers, as well as other enteric infections. For example, strategies to identify vaccines that activate T_eff_ without the concomitant activation of suppressive T_reg_ responses, or that elicit an optimal balance between T_eff_ and T_reg_ responses may result in improved protective efficacy.

## Materials and Methods

### Volunteers and isolation of peripheral blood mononuclear cells (PBMC)

Healthy adult volunteers aged 18–60 were recruited by the Centre for Clinical Vaccinology and Tropical Medicine, Oxford, UK, to participate in this study. Volunteers with history of typhoid fever or immunization against typhoid fever were excluded [7]. Volunteers were orally challenged with 1–5x10^4^ CFU of wt-*S*. Typhi (Quailes strain) suspended in sodium bicarbonate at Oxford University in compliance with the National Research Ethic Service (NRES), Oxford Research Ethics Committee A [7]. Close monitoring was performed throughout the study, and at the time of typhoid fever diagnosis (TD, as determined by blood culture-confirmed *S*. Typhi bacteremia or development of a fever ≥38°C for ≥12 hours), volunteers were treated with a 2-week course of antibiotics (Ciprofloxacin, 500mg twice daily). Those volunteers who did not developed typhoid fever (No TD) received a 2-week course of antibiotics at day 14 post-challenge. PBMC collected from 12 randomly selected volunteers (TD n = 6, No TD n = 6) participating in the challenge trial were used in this study. Selection was made based on the number of available PBMC with those volunteers having more PBMC utilized for the studies. PBMC were isolated prior to challenge and at 9–11 time-points following challenge (**S1 Fig**). Isolation was performed by Lymphoprep gradient centrifugation (Axis-Shield, Oslo, Norway) and PBMC were cryopreserved in liquid nitrogen following standard techniques within four hours of initial blood draw. Viability of cryopreserved PBMC was assessed after thawing of cells and an overnight rest at 37°C with 5% CO_2_ (as described in *ex vivo* stimulation).

### Target/stimulator cells

B-LCL were generated from autologous PBMC for each volunteer as previously described [45]. Briefly, B-LCL were established using supernatant from the B95.8 cell line (ATCC CRL1612; American Type Culture Collection) as the source of EBV. PBMC from each volunteer were incubated with EBV containing supernatant and cyclosporine (0.5 μg/mL; Sigma, St. Louis, MO) at 37°C with 5% CO_2_ for 2–3 weeks. B-LCL were maintained in culture or cryopreserved until use.

### Infection of target/stimulator cells

Target cells were infected by incubation with wild-type *S*. Typhi strain ISP1820 in RPMI 1640 media (Gibco, Carlsbad, CA) without antibiotics for 3 hours at 37°C with 5% CO_2_ as previously described [45]. On the day following infection the cells were gamma irradiated (6000 rad). To confirm that targets were infected with *S*. Typhi, cells were stained with anti-*Salmonella* common structural Ag (CSA-1)-FITC (Kierkegaard & Perry, Gaithersburg, MD) and analyzed by flow cytometry on an LSRII flow cytometer (BD Biosciences, San Jose, CA) [8]. The percentage of cells infected with *S*. Typhi was recorded for each experiment and the infected targets were only used if infection rates were >30% of viable cells.

### Ex vivo stimulation

PBMC were thawed and rested overnight at 37°C. Cells were then resuspended in RPMI 1640 media (Gibco) supplemented with 100 U/mL penicillin (Sigma), 100 μg/mL streptomycin (Sigma), 50 μg/mL gentamicin (Gibco), 2 mM L-glutamine (Gibco), 2.5 mM sodium pyruvate (Gibco), 10 mM HEPES buffer (Gibco), and 10% fetal bovine serum (Gemini Bioproducts, West Sacramento, CA) at a concentration of 1x10^6^ cells/mL in sterile 5 mL round bottom tubes (BD Falcon, Franklin Lakes, NJ). PBMC were stimulated with *S*. Typhi-infected B-LCL or B-LCL alone (negative control). After 2 hours, Golgi Stop (containing monensin) and Golgi Plug (containing brefeldin A) from BD were added at concentrations of 0.5 μl/mL and cultures continued overnight at 37°C in 5% CO_2_. Media alone was used as an additional negative control.

### Conventional flow cytometric analyses

Following stimulation as described above, cells were plated in 96-well V-bottom plates for staining. Cells were washed once with staining buffer (phosphate buffered saline with 0.5% BSA and 0.1% sodium azide) and stained for live/dead discrimination using Invitrogen LIVE/DEAD fixable yellow dead cell stain kit (Invitrogen, Carlsbad, CA). Fc receptor blocking was performed with human immunoglobulin (Sigma; 3 μg/mL) followed by surface staining, performed as previously described.[8] Briefly, cells were surface stained with panels that included the following fluorochrome-conjugated monoclonal antibodies against: CD14-BV570 (M5E2, Biolegend, San Diego, CA), CD19-BV570 (HIB19, Biolegend), CD3-BV650 (OKT3, Biolegend), CD4-APC-H7 (RPA-T4, BD), CD25-PECy7 (M-A251, BD), CCR6/CD196-PE (11A9, BD), HLA-DR-Qdot 800 (Life technologies, Grand Island, NY), integrin α4β7-Alexa 647 (clone ACT-1, conjugated in-house), CXCR3/CD183-Alexa 700 (1C6/CXCR3, BD), LFA-1/CD11a-Alexa 488 (HI111, Biolegend), NRP-1/CD304-APC (12C2, Biolegned), CD27-BV605 (4S.B3, Biolegend), CD39-BV421 (A1, Biolegend), and Tim-3-Alexa 700 (344823, R&D, Minneapolis, MN) at 4°C for 30 minutes. The cells were then fixed and permeabilized using FoxP3 IC fixation and permeabilization buffers from eBiosciences according to manufacturer’s recommendations. Intracellular staining with FoxP-PerCP-Cy5.5 (236A/E7, BD), CTLA-4/CD152-PECy5 (BNI3, BD), PD-1/CD279-BV421 (EH12.1, BD) and Ki67-BV605 (Ki67, Biolegend) was performed for 20 minutes at room temperature. After staining, cells were fixed in 1% paraformaldehyde and stored at 4°C until analyzed. Flow cytometry was performed using a customized LSRII flow cytometer (BD). Flow cytometry data were analyzed using WinList version 7 (Verity Software House, Topsham, ME) software package. Graphs were generated using GraphPad Prism version 6 (Graphpad Software, San Diego, CA).

### CD25-depletion studies

In a subset of volunteers, CD25 cells were depleted- or mock-depleted using anti-CD25 or pan anti-mouse IgG Dynabeads, respectively (Invitrogen) as previously described [25]. Briefly, thawed PBMC were rested overnight as described above. Following the overnight rest, PBMC were divided into two aliquots consisting of 2.1–3 x 10^6^ cells and either mock-depleted or depleted of CD25 cells using magnetic bead separation. Depleted (mock and CD25) PBMC were stimulated with *S*. Typhi-infected B-LCL or non-infected B-LCL (negative control) as described above.

### Mass cytometry

Following CD25- or mock-depletion and stimulation with *S*. Typhi-infected B-LCL, cells were stained for mass cytometry with a panel of 22 metal-conjugated mAb to detect both T_reg_ and responder T cells. A table of the mAb used is shown in supplementary materials (**S1 Table**). Viability staining was performed with cisplatinum (Sigma; 25 μM) for 60 seconds. Following cisplatinum, samples were Fc-blocked with human immunoglobulin (Sigma; 3 μg/mL) followed by surface staining, performed as previously described. Fixation and permeabilization were performed with FoxP3 IC fixation and permeabilization buffers (eBiosciences) followed by intracellular staining. Samples were stained with an Ir^191/193^ DNA intercalator for cell detection by mass cytometry within 48 hours of sample acquisition and re-suspended in EQ4 normalization beads (Fluidigm, Sunnyvale, CA). Acquisition was performed using a CyTOF mass cytometer (Fluidigm, formerly DVS Sciences). Data were analyzed with Fluidigm Cytobank.

### Statistical analyses

Observations were grouped by day following challenge in the following periods: pre-challenge, days 1 to 4, days 5 to 9, days 10 to 14, and days 21 to 28 (there were no observations between days 14 and 21). Volunteers often contributed more than one observation to these time periods. To compare mean values by time period and group, while accounting for the lack of independence between multiple measures from the same volunteer at the same time period and across time periods, we used mixed effects models, including a random effect for subject, fit by restricted maximum likelihood. Through simulation experiments we confirmed that this approach provided valid statistical inference for data sets of this size.

### Ethics statement

The human challenge study was performed in compliance with the National Research Ethic Service (NRES), and approved by the Oxford Research Ethics Committee A. All volunteers provided written informed consent.

## Supporting Information

S1 Fig Outline of study design. Volunteers were challenged with wild-type *S*. Typhi on day 0. PBMC were obtained prior to challenge and at up to 10 time-points after challenge.
(TIF)

S2 Fig Gating strategy for identification of T_reg_ homing potential and activation. Lymphocytes were gated on forward versus side scatter, followed by exclusion of doublets. Yellow Viability Dye live/dead staining was used to exclude dead cells and anti-CD14 and CD19 were used to exclude monocytes and B cells, respectively with positive selection of CD3+ T cells. CD4+ FoxP3+ cells were identified followed by gating on CTLA-4 and CD25. T_reg_ were defined as CD4+ FoxP3+ CTLA4+ CD25+. Representative histograms depicting the expression of homing molecules integrin α4β7, CXCR3, and CCR6, as well as the expression of activation molecules HLA-DR, PD-1, CD27, LFA-1, NRP-1, CD39, and Tim-3.
(TIF)

S3 Fig *S*. Typhi-specific activation of circulating T_reg_. Net *S*. Typhi-specific modulation of the expression of **A**) CD39, (TD n = 3, No TD n = 3) and **B**) Tim-3, (TD n = 5, No TD n = 6) on T_reg_. Values are shown as the mean +/- SEM. Statistically significant differences in the modulation of the expression of CD39 between pre- and days 10–14 post-challenge in TD volunteers (#) are indicated. TD (blue squares); No TD (red circles). Values from multiple time-points were grouped together in time segments (1–4, 5–9, 10–14, and 21–28 days post-challenge) to account for variability in the samples available from each volunteer. Some volunteers had samples from multiple time-points in a time-segment resulting in more data points than the corresponding number of volunteers.
(TIF)

S1 Table Monoclonal antibodies used for mass cytometry staining. (XLSX)
