# Supplementary figures and images for: Activation of Salmonella Typhi-Specific Regulatory T Cells in Typhoid Disease in a Wild-Type S. Typhi Challenge Model
Source: PLoS Pathog. 2015 May 22;11(5):e1004914. doi: 10.1371/journal.ppat.1004914 (PMC4441490; doi:10.1371/journal.ppat.1004914)

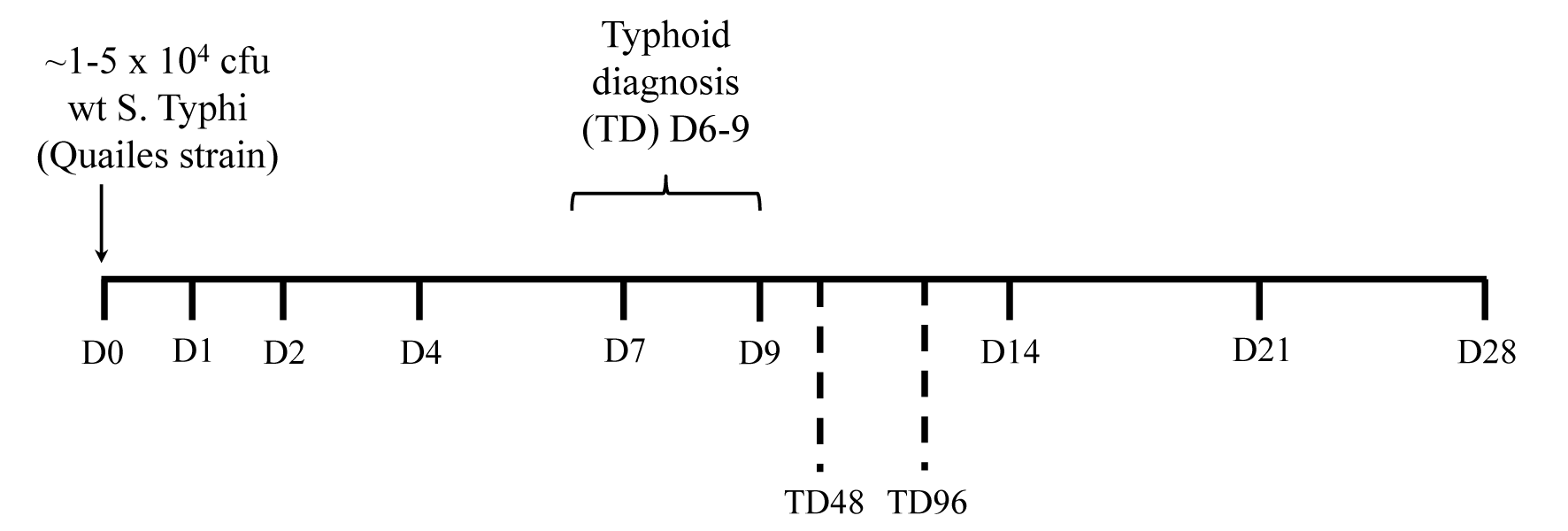

Supplement: S1 Fig — Volunteers were challenged with wild-type S. Typhi on day 0. PBMC were obtained prior to challenge and at up to 10 time-points after challenge. (TIF) [file ppat.1004914.s001.tif]

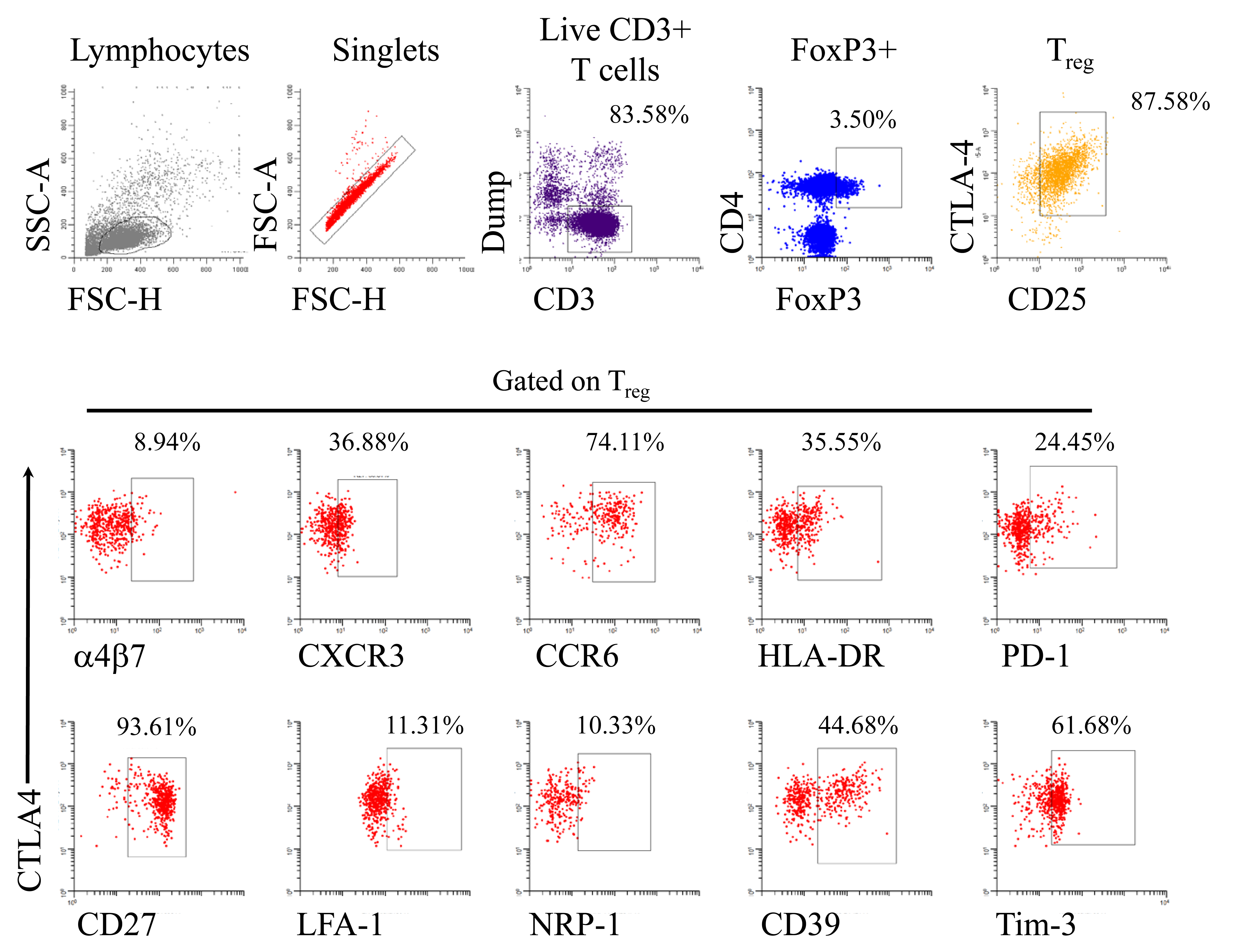

Supplement: S2 Fig — Lymphocytes were gated on forward versus side scatter, followed by exclusion of doublets. Yellow Viability Dye live/dead staining was used to exclude dead cells and anti-CD14 and CD19 were used to exclude monocytes and B cells, respectively with positive selection of CD3+ T cells. CD4+ FoxP3+ cells were identified followed by gating on CTLA-4 and CD25. Treg were defined as CD4+ FoxP3+ CTLA4+ CD25+. Representative histograms depicting the expression of homing molecules integrin α4β7, CXCR3, and CCR6, as well as the expression of activation molecules HLA-DR, PD-1, CD27, LFA-1, NRP-1, CD39, and Tim-3. (TIF) [file ppat.1004914.s002.tif]

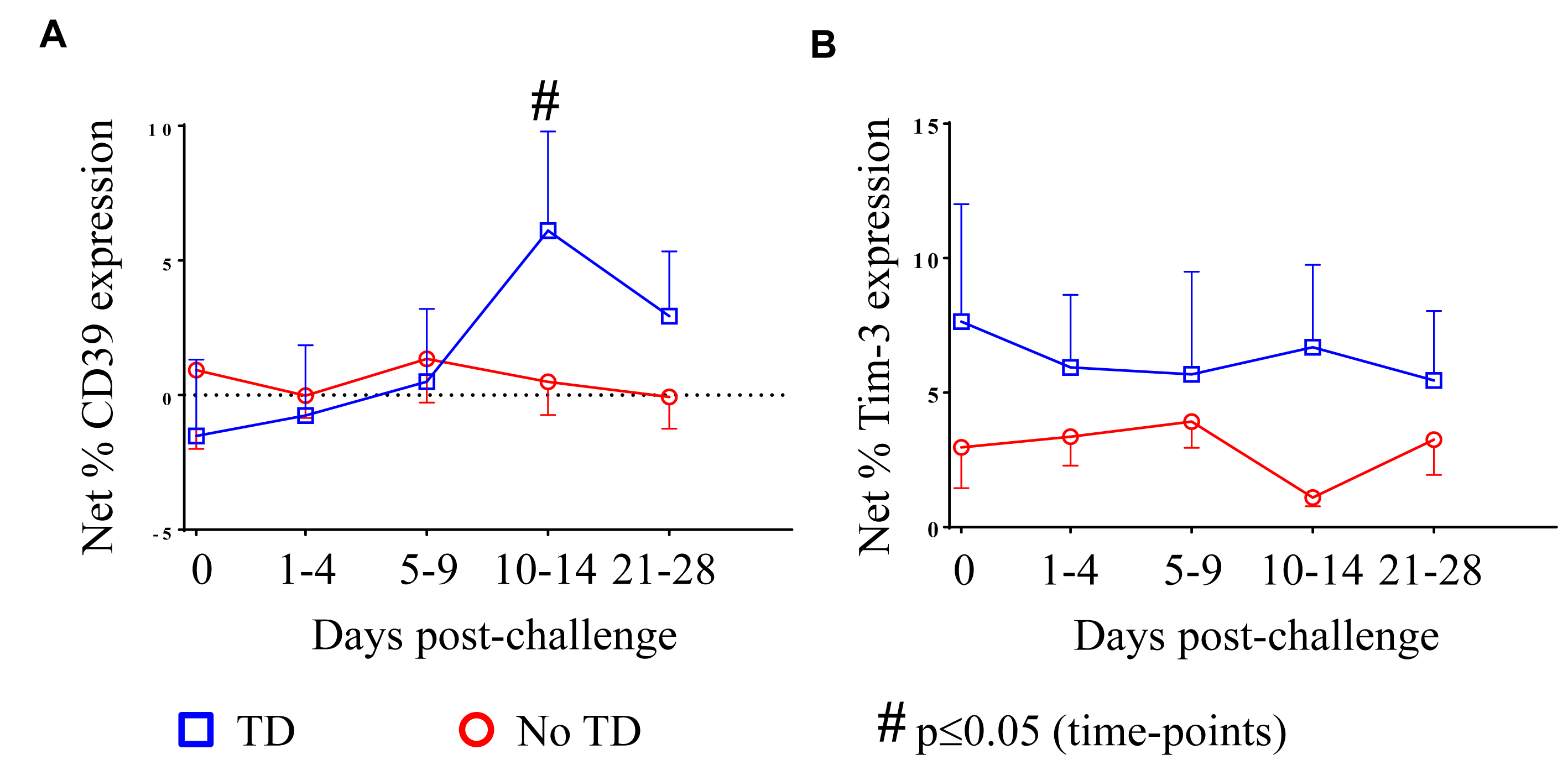

Supplement: S3 Fig — Net S. Typhi-specific modulation of the expression of A) CD39, (TD n = 3, No TD n = 3) and B) Tim-3, (TD n = 5, No TD n = 6) on Treg. Values are shown as the mean +/- SEM. Statistically significant differences in the modulation of the expression of CD39 between pre- and days 10–14 post-challenge in TD volunteers (#) are indicated. TD (blue squares); No TD (red circles). Values from multiple time-points were grouped together in time segments (1–4, 5–9, 10–14, and 21–28 days post-challenge) to account for variability in the samples available from each volunteer. Some volunteers had samples from multiple time-points in a time-segment resulting in more data points than the corresponding number of volunteers. (TIF) [file ppat.1004914.s003.tif]
